# Involvement of family caregivers in dementia care research: a scoping review protocol

**DOI:** 10.1186/s13643-024-02696-w

**Published:** 2024-11-11

**Authors:** Franziska Anushi Jagoda, Julian Hirt, Claudia Mueller, Margareta Halek

**Affiliations:** 1 Faculty of Health, School of Nursing Science, Witten/Herdecke University, Alfred-Herrhausen-Strasse 50, Witten, 58455 Germany; 2 Department of Health, Center for Dementia Care, Eastern Switzerland University of Applied Sciences, Rosenbergstrasse 59, St. Gallen, 9000 Switzerland; 3 Department of Clinical Research, University of Basel and University Hospital Basel, Spitalstrasse 2, Basel, 4031 Switzerland; 4 Institute for Information Systems, University of Siegen, Kohlbettstrasse 15, Siegen, 57072 Germany

**Keywords:** Nurse, Nursing, Family caregiver, Dementia, Involvement, Research

## Abstract

**Background:**

Family caregivers of people with dementia are a distinct group due to the particularly stressful and time-intensive care situation at home. Despite these challenges, involving them in research is crucial to better understand and address their specific needs. However, little evidence exists regarding a tailored approach for researchers for this group considering their situation at home.

**Methods:**

A scoping review will be conducted following the Joanna Briggs Institute methodological guidance, including the databases MEDLINE (PubMed), CINAHL, Scopus (Elsevier), and PsycINFO (EBSCO). The review will include family caregivers of people with dementia, regardless of age, gender, or ethnicity, who have been actively involved in research throughout the research process. Moreover, sources of evidence from any country in both English and German are eligible for inclusion. Sources will be screened by two independent reviewers. Results will be extracted using a tailored charting tool and presented in the final report according to the research questions and objectives.

**Discussion:**

Developing a tailored approach to involve family caregivers of people with dementia in research and development has profound importance to both the scientific community and the target group itself.

**Systematic review registration:**

Open Science Framework 10.17605/OSF.IO/PMZYV.

**Supplementary Information:**

The online version contains supplementary material available at 10.1186/s13643-024-02696-w.

## Background

A democratic way to generate added value in health research is participatory research [1]. This added value is reflected in proximity to everyday life and an increase in outcome relevance for both patients and those who care for them. Involvement in the health research context is a strongly value-based process to promote democracy, social justice, and human rights in the context of research. In a participatory process, individual preferences and joint decisions are reconciled [2–4]. INVOLVE (est. 1996, UK) defines “public involvement” as “[…] research being carried out ‘with’ or ‘by’ members of the public rather than ‘to’, ‘about’ or ‘for’ them” [5]. Indeed, the role of people involved goes beyond the role of a mere research object. Rather, the relationship between researchers and co-researchers is based on partnership where research is designed together.

Levels of involvement in health research vary: target groups can be engaged on an advisory board, in contributing to research questions, or even conducting research tasks within the study independently [6]. After being involved in research, people report feeling empowered, that they have acquired new skills such as interviewing, or have gained confidence in speaking and improved their knowledge about research [1]. But for the target group, involvement in health research usually goes hand in hand with both increased time expenditure and increased cognitive effort. As a result, tailored approaches for specific populations are useful [6].

Many international health organizations such as the Canadian Institute of Health Research [7] and the American independent nongovernmental organization Patient-Centered Outcomes Research Institute advocate for involvement in research [8]. However, it is unclear how involvement in research can work profitably for specific target groups and for the researchers. Furthermore, it is particularly questionable to include groups that are already heavily involved in the health care of others [9–11].

Research suggests that the priorities of family caregivers of people with dementia have been overlooked in the development of services to support them [12, 13]. Ceci and Symonds Brown [14] go further, arguing that researchers fail to look at the person with dementia and their family caregiver on a deeper level, resulting in a lack of understanding behind their care arrangement. This limited perspective can produce narrow understandings of the arrangement, and “[…] intervention research tends to emerge as detached from the life in which it is lived […]” [14]. Moreover, needs in the care context vary according to the diagnosis of the person in need of care and changing roles and situations in families. Therefore, interventions should not be developed in isolation from the person in need of care and the care arrangement. Rather, the conditions and limitations of home care should also be considered, thus requiring greater involvement of family caregivers in research and development [15].

Previous research has shown that caregiving for people with dementia is associated with great burden due to the severity of cognitive and behavioral impairments and the duration of the care period [16]. Behavioral changes such as aggression or agitation are particularly stressful for family caregivers of people with dementia [17]. In addition, these family caregivers typically have even less time for themselves while caring for the person’s activities of daily living, balancing caregiving with their existing lifestyle and responsibilities [18]. The question therefore arises as to how such a group can also be involved in research for its own benefit and at the same time actively participate in shaping research.

In the field of dementia care research, there is a growing interest in participatory research that focuses on the care and the physical, cognitive, psychological, and emotional support of people with dementia and their caregivers. However, there seem to be only few scientific studies reporting on research participation with family caregivers of people with dementia. For example, O'Connell and Crossley [19] developed a video conferencing system with family caregivers to provide support in rural areas, while Tamagnini and Cotton [20] collaborated with a group of family caregivers to produce a film on research involving animals in dementia research. Only a few studies also methodologically address the issue of participation of family caregivers of people with dementia in research. Poland and Charlesworth [21] report that there were implicit expectations between participants in the participatory process, or the co-researchers involved in the data analysis had a different approach and opinion on the interviews conducted, and these differences led to discussions. Nevertheless, family caregivers are clear in their assertion that they have particular input to bring to research [22].

The question therefore arises as to whether family caregivers’ needs in this context are known, the extent of time that family caregivers of people with dementia can and want to commit, and how these aspects can be met in a participatory process. Considering that family caregivers are a special group with limited time resources, a discussion for an optimal way of involvement seems fundamentally important. Due to the growing interest in participatory research of family caregivers of people with dementia, roles, responsibilities, tasks, and roles need to be clarified, and barriers need to be identified [23]. This may guide researchers with evidence-based information on orienting their research towards caregivers [6, 24–26].

This leads us to the objective to answer the following research question: What is known about the extent, range, and nature of research activity in the area of participatory dementia care research involving family caregivers?

The three sub-questions are as follows:

1. What strategies have been used by the research team to involve family caregivers of people with dementia in research?
2. What roles, barriers, and enablers are being reported to involve family caregivers of people with dementia in research?
3. What impact can the researchers and family caregivers of people with dementia describe as a result of the research involvement?

## Methods/design

To identify and map the evidence on the involvement of family caregivers in dementia care research, we have chosen a scoping review design guided by the Joanna Briggs Institute (JBI) methodology [27]. To structure our report, we will use the Preferred Reporting Items for Systematic reviews and Meta-Analyses extension for Scoping Reviews (PRISMA-ScR) [28] in addition to the Preferred Reporting Items for Systematic review and Meta-Analyses for Protocols (PRISMA-P) 2015 checklist [29].

This protocol is registered with the Open Science Framework: 10.17605/OSF.IO/PMZYV.

### Inclusion criteria

Table 1 shows the inclusion and exclusion criteria.

**Table 1:** Inclusion/exclusion criteria

Domain	Inclusion	Exclusion	Reason
*P*articipants *Study population*	Family caregivers of people with dementia (e.g., partners, children, friends, neighbors)	Family caregivers of people with conditions other than dementia	Focus on dementia
	All forms and severities of dementia		
*C*oncept *Intervention*	Research involvement as an active research partner throughout the research cycle (preparatory phase, execution phase, and translational phase)	Research involvement as a research subject	
*C*ontext *Setting*	Research related to dementia care	Research not related to dementia care, i.e., research related to biomedical and genetic dementia research	Results are supposed to focus on involvement from within the dementia-specific home care setting
	Community home setting	Institutional care setting (e.g., nursing or care home)	
Sources of evidence	Empirical study design (i.e., qualitative, quantitative, and mixed-methods design, irrespective of study size)	Evidence synthesis	
	Original research as indicated by an introduction, methods, results, and discussion (IMRaD) structure including study protocols, corrigenda, and errata	Opinion pieces (e.g., commentary, editorial)	
	Journal articles	Other publication format and gray literature (e.g., thesis, book, Internet report)	
	German and English language	Other languages	
	No restriction of publication year		
	No restriction of country of study conduct		

#### Participants

We will consider studies that involve family caregivers of people with dementia, regardless of age, gender, ethnicity, dementia form, or dementia severity. “Family caregiver” refers to both informal caregivers (partners and children of the person with dementia) and helpers such as friends or neighbors. We will exclude studies that involve family caregivers of people or patients with lived experience of a condition other than dementia.

#### Concept

The concept to be examined in this scoping review is involvement in research [5, 7, 8]. International definitions are consistent as to including individuals with personal experience, such as patients, family, or friends while forming an active and meaningful partnership that shapes research [5, 8]. Involvement may therefore occur throughout the research cycle and could include, but is not limited to, identifying research priorities, undertaking data collection, or being a member of an advisory board [5]. According to Shippee and Domecq Garces [30] and their framework for user engagement, there are three different phases in research in which involvement can take place: the preparatory phase, the execution phase, and the translational phase, each with different subphases. Studies will be excluded if they do not provide enough detail to ascertain whether family caregivers of people with dementia were engaged beyond the role of research subjects.

#### Context

Our focus will be on dementia care research, specifically on the care and physical, cognitive, psychological, and emotional support of people with dementia and their caregivers, in which family caregivers of people with dementia have been involved in the research process. This includes studies of any empirical study design and size, in which family caregivers of people with dementia were involved in planning, conducting, or disseminating research. The research process can, therefore, take place in the community, at the families’ homes, or at specific institutions, like universities or research facilities. Sources of evidence from any country will be included. As this scoping review aims at results regarding involvement from within the home and community care setting, studies focusing on research conducted in and focusing on the nursing home environment will be excluded. In addition, studies assessing engagement in treatment and healthcare and not in the research process will be excluded.

#### Sources of evidence

The study will include all dementia-care-focused research articles that have an empirical study design, are published in an academic journal, and report on original research (primary or secondary data analysis) as indicated by an introduction, methods, results, and discussion structure (IMRaD) and related errata. Evidence syntheses, dissertations, theses, commentaries, and editorials will be excluded. We will consider articles published in English and German language with no restrictions on the publication year.

#### Search strategy

The search strategy will follow the three-step search strategy recommended by the JBI [31]. At first, an initial, limited search in MEDLINE (PubMed) was conducted to identify relevant articles within the scope of the research questions and key concepts. This step identified relevant keywords from the abstract, title, and index words describing the articles. As a second step, a complete search using all identified keywords will be executed in all databases (see the “Information sources”). The reference lists of all identified eligible articles and reports will be searched for additional sources using Scopus. F. J. designed the search strategy which was reviewed by J. H. using PRESS (Peer Review of Electronic Search Strategy) [32]. An outline of the final search strategy for MEDLINE is detailed in Table 2.

**Table 2:** Search strategy MEDLINE via PubMed

Search component	String
Family caregiver	(famil*[TIAB] OR spouse*[TIAB] OR relative*[TIAB] OR informal[TIAB] OR household*[TIAB] OR dyad*[TIAB] OR caregiv*[TIAB] OR "care giver"[TIAB] OR carer*[TIAB] OR Family[MeSH] OR Caregivers[MeSH])
	AND
Dementia	(dement*[TITLE] OR Alzheimer*[TITLE] OR dementia[MeSH])
	AND
Involvement	(involv*[TITLE] OR PPI[TITLE] OR engag*[TITLE] OR participat*[TITLE] OR "co-produc*"[TITLE] OR "co-design*"[TITLE] OR collaborat*[TITLE] OR cooperat*[TITLE] OR "emancipatory"[TITLE] OR "user-led"[TITLE] OR "action research"[TITLE] OR "advisory group*"[TITLE] OR consult*[TITLE] OR panel*[TITLE] OR partner*[TITLE] OR "experts by experience"[TITLE] OR "citizen science"[TITLE] OR Community-Based Participatory Research[MeSH] OR Patient Participation[MeSH] OR Citizen Science[MeSH])

#### Information sources

The electronic databases MEDLINE (PubMed), CINAHL, Scopus (Elsevier), and PsycINFO (EBSCO) will be searched. Pertinent evidence syntheses identified through the database search are kept for identification of additional primary studies. We will also include articles found through expert advice. Experts are identified by searching the relevant organizations and will be matched with the authors of the identified papers. These experts are then contacted to identify additional references.

#### Study selection

All identified references will be collected and stored in EndNote X9, and duplicates will be removed (Clarivate Analytics, PA, USA). The study screening will be undertaken in Rayyan [33]. Subsequently, a randomly selected 20% of titles, abstracts, and full texts will be screened by two independent reviewers (F. J. & J. H.) for eligibility. The selection of the remaining references will be made by one reviewer (F. J.). Abstracts that do not explicitly indicate that family caregivers of people with dementia were involved will be excluded. Reasons for exclusion of references on the full-text level will be reported in an appendix of the final scoping review report. Disagreements between the reviewers (independent screening) and uncertainties during single-reviewer screening will be resolved through discussion or with another reviewer (J. H. or M. H.). The results of the literature selection process will be reported in full in the final report and will be presented in a Preferred Reporting Items for Systematic reviews and Meta-Analyses for Scoping Reviews (PRISMA-ScR) flow diagram.

#### Data extraction

The research team jointly developed a data extraction sheet. Papers included in the review will be extracted according to the sheet containing details about the participants, the concept, and context, along with additional meta-information such as journal and year of publication following the JBI guideline. The data extraction sheet is based on three sets of criteria: (1) the JBI Manual for Evidence Synthesis for data extraction in scoping reviews [34], (2) on a recent methodical paper by Pollock and Peters [35], and (3) on the Guidance for Reporting Involvement of Patients and the Public (GRIPP2) [36]. The sheet also focuses on the review objective and research questions. A first draft of the extraction tool is provided in Additional file 1: Appendix I. One reviewer (F. J.) will identify and extract the relevant information using the data extraction tool. Ten percent of the extractions will be double-checked by another reviewer (J. H.) to ensure that relevant data has been extracted. The data extraction tool will be iteratively updated and modified during the charting process of included sources when necessary [35].

#### Data analysis and presentation

The charted data will be presented descriptively using tables and figures. Meta-information such as year or period of publication and country of origin will also be displayed. A framework for user engagement project phases developed by Shippee and Domecq Garces [30] will be used to identify and categorize the research phases, where involvement takes place. Additionally, the framework will be used to describe the roles, barriers, and enablers within these phases. The preparatory phase consists of identifying research priorities, whereas the execution phase involves the development and carrying out the research design. Recruitment and data analysis and collection also fall into this phase. In the third phase, the translational phase, dissemination, and implementation of the research findings occur. Finally, this framework was developed using 37 sources describing conceptualizations of patient and service-user engagement [30].

A narrative summary will support the tables and figures to relate it to the scoping review’s objective and research questions. The findings will be discussed in the broader perspective of research practice.

#### Consultation

This step is considered to be of vital importance for any topic regarding involvement in research, as it helps researchers to see their results from the perspective of different stakeholders [37]. Once the preliminary results are finalized, we will consult four family caregivers of people with dementia to present the results to them in a focus group interview. The focus group can take place at the location chosen by the group: either in the community, at home, or via video chat. The family caregivers can become involved via local dementia networks or through attending dementia groups visited by the researchers. We will present main themes and seek family caregivers’ opinions, as well as assess the importance of each theme. Themes will be rated by family caregivers on a 3-point scoring scale (“Not particularly important,” “Important,” and “Very important”), in accordance with Morbey and Harding [38]. Next, the researchers will facilitate discussions around the ratings. The family caregivers’ feedback and interpretations will help us understand the results of the scoping review in more depth. Comments and ratings will be noted in written form. Focus group results will be presented as an additional section in the final scoping review paper.

## Discussion

The scoping review will focus on the involvement of family caregivers of people with dementia in research. Since stakeholder engagement in research is a timely topic, this scoping review will show how widespread the inclusion of family caregivers is in dementia care research and explore methods used in existing studies. Moreover, the review will describe the experiences of families and researchers with the involvement process and will outline the impact of such involvement. This can help researchers plan and conduct their studies in a targeted manner. It can therefore be useful to improve the appropriateness and inclusivity of research that targets the everyday life of family caregivers of people with dementia at home. In this context, it can be assumed that the included studies show a high heterogeneity in terms of methodologies and approaches, while they may also come from different disciplines [10]. This challenge will be addressed using a standardized data extraction template and discussion within the review team if conflicting aspects arise.

Family caregivers of people with dementia are a very special group with characteristics that do not necessarily make involvement in dementia care research easier. However, since they play a vital role in the care of people with dementia at home and are experts in the care situation, their involvement in research and projects is essential. Furthermore, the outcome of these projects will have a lasting impact on home care. In addition, funding agencies increasingly demand participation in research. Family caregivers can bring their implicit knowledge into dementia care research, which could lead to results that are more relevant to their everyday life. As this group faces unique challenges not only with regard to time constraints and responsibilities, a tailored approach to their involvement in research is critical.

## Supplementary Information

Additional file 1: Appendix I. Data extraction tool. Table 1: Citation details. Table 2: Research characteristics. Table 3: Involvement characteristics. Table 4: Family caregiver’s reflection of involvement. Table 5: Researchers’ reflection of family caregiver’s involvement. Table 6: Impacts of involvement.

## Data Availability

N.A.

## Abbreviation

JBI: Joanna Briggs Institute
