# Supplementary material for: Involvement of family caregivers in dementia care research: a scoping review protocol
Source: Syst Rev. 2024 Nov 11;13:277. doi: 10.1186/s13643-024-02696-w (PMC11552150; doi:10.1186/s13643-024-02696-w)
Supplement: Supplementary file 1 — Additional file 1: Appendix I. Data extraction tool. Table 1: Citation details. Table 2: Research characteristics. Table 3: Involvement characteristics. Table 4: Family caregiver’s reflection of involvement. Table 5: Researchers’ reflection of family caregiver’s involvement. Table 6: Impacts of involvement. [file 13643_2024_2696_MOESM1_ESM.docx]

**Appendix I**

**Data extraction tool**

Table 1: Citation details

| **#Citation** | **First author(s) surname** | **Year of Publication** | **Journal** | **Title** | **Country of study conduct** |
| --- | --- | --- | --- | --- | --- |
|  |  |  |  |  |  |

Table 2: Research characteristics

| **#Citation** | **Aim** | **Design** |
| --- | --- | --- |
|  |  |  |

Table 3: Involvement characteristics

| **#Citation** | **Who was engaged?** | **Total number of people involved** | **Geographical location (from which family caregivers were recruited)** | **Research cycle phase (Shipee et al., 2015)** | **Definition of involvement** |
| --- | --- | --- | --- | --- | --- |
|  |  |  |  |  |  |
|  | **Types of involving activity** | **Frequency of involvement** | **Roles adopted** | **Type of compensation** |  |
|  |  |  |  |  |  |

Table 4: Family caregiver’s reflection of involvement

| **#Citation** | **Barriers** | **Enablers** | **Overall perception of the involvement** |
| --- | --- | --- | --- |
|  |  |  |  |

Table 5: Researchers’ reflection of family caregiver’s involvement

| **#Citation** | **Barriers** | **Enablers** | **Overall perception of the involvement** |
| --- | --- | --- | --- |
|  |  |  |  |

Table 6: Impacts of involvement

| **#Citation** | **Definition of Impact** | **Impact reported (researchers)** | **Measurement** | **Impact reported (family caregivers)** | **Measurement** |
| --- | --- | --- | --- | --- | --- |
|  |  |  |  |  |  |
